# The presence of cardiotropic viral genomes is not increased in atrial tissue of atrial fibrillation patients

**DOI:** 10.1007/s12471-022-01660-4

**Published:** 2022-01-31

**Authors:** L. Wu, R. W. Emmens, J. van Wezenbeek, W. Stooker, C. P. Allaart, A. B. A. Vonk, A. C. van Rossum, K. C. Wolthers, H. W. M. Niessen, P. A. J. Krijnen

**Affiliations:** 1 Department of Pathology, Amsterdam University Medical Centre, location VUmc and AMC, Amsterdam, The Netherlands; 2 Amsterdam Cardiovascular Sciences, Amsterdam, The Netherlands; 3 Department of Cardiac Surgery, Amsterdam University Medical Centre, location VUmc, Amsterdam, The Netherlands; 4 Department of Virology, Amsterdam University Medical Centre, location AMC, Amsterdam, The Netherlands; 5 Department of Cardiac Surgery, OLVG, Amsterdam, The Netherlands; 6 Department of Cardiology, Amsterdam University Medical Centre, location VUmc, Amsterdam, The Netherlands

**Keywords:** Atrial fibrillation, Inflammation, Parvovirus B19

## Abstract

**Background:**

Infections with potentially cardiotropic viruses are associated with the development of atrial fibrillation (AF). However, whether direct viral infection of the atria is involved in the pathogenesis of AF is unclear. We have therefore analysed the presence of cardiotropic viral genomes in AF patients.

**Methods:**

Samples of left atrial tissue were obtained from 50 AF patients (paroxysmal, *n* = 20; long-standing persistent/permanent, *n* = 30) during cardiac surgery and from autopsied control patients (*n* = 14). Herein, the presence of PVB19, EBV, CMV, HHV‑6, adenovirus and enterovirus genomes was determined by polymerase chain reaction. The densities of CD45+ and CD3+ cells and fibrosis in the atria were quantified by (immuno)histochemistry.

**Results:**

Of the tested viruses only the PVB19 genome was detected in the atria of 10% of patients, paroxysmal AF (2 of 20) and long-standing persistent/permanent AF (3 of 30). Conversely, in 50% of controls (7 of 14) PVB19 genome was found. No significant association was found between PVB19 and CD45+ and CD3+ cells, or between the presence of PVB19 and fibrosis, in either control or AF patients.

**Conclusion:**

The presence of viral genomes is not increased in the atria of AF patients. These results do not support an important role for viral infection of the atria in the pathogenesis of AF.

**Supplementary Information:**

The online version of this article (10.1007/s12471-022-01660-4) contains supplementary material, which is available to authorized users.

## What’s new?

- Of the tested viruses, only the genome of parvovirus B19 (PVB19) was detected in 10% of atrial fibrillation (AF) patients but in 50% of control patients.
- There was no significant association between atrial inflammation and the presence of the PVB19 genome in either control or AF patients.
- The atrial viral infection is unlikely to contribute to AF development.

## Introduction

Atrial fibrillation (AF) is the most common cardiac arrhythmia and is a major cause of stroke, cardiovascular morbidity and sudden death [1]. Although the exact cause of AF is often not clear, certain underlying conditions predispose toward AF development, including ageing, hypertension, diabetes, obesity as well as heart, valve and coronary artery disease. AF progression is associated with structural atrial remodelling, typified by increased atrial fibrosis and fatty infiltration, which disrupts electrical signal conduction, thus facilitating atrial arrhythmia. Inflammation may be a key driver of this process and inflammatory cell infiltration is indeed commonly observed in the atria of AF patients [2, 3].

Viral infection may be another trigger for AF development. Infections with multiple viruses, including influenza virus, hepatitis viruses, human immunodeficiency virus and human herpes viruses, have been shown to be significantly associated with AF development [3–7]. Moreover, the increased expression of Toll-like receptor 2 on monocytes [8] and high enrichment of viral response genes in lymphocytes [9], which have been found in AF patients, suggest an involvement of (chronic) viral infection in AF pathogenesis. However, whether this is the result of systemic inflammation that occurs with these infections and/or direct infection of the atria is unknown.

Interestingly, many of these viruses are cardiotropic and their genome has been found in the ventricles of myocarditis patients [10]. In mice with coxsackievirus B3-induced myocarditis, viral genomes and inflammation were also found in the atria [11] and ventricular myocarditis coincided with atrial myocarditis in patients [2]. This suggests that cardiotropic viruses may directly infect the atria and thereby be involved in the pathogenesis of AF.

We therefore analysed the presence of cardiotropic viral genomes in left atrial tissue of patients with different forms of AF [1], e.g. paroxysmal, long-standing persistent and permanent AF, collected during heart surgery and of control patients.

## Materials and methods

### Patients

Left atrial auricle tissue was obtained from AF patients (paroxysmal, *n* = 20; long-standing persistent and permanent, *n* = 30) who underwent open-heart surgery at the OLVG Hospital in Amsterdam. The tissue was taken at the start of the surgery. As a control, left atrial tissue from patients (*n* = 14) without AF or any other form of heart disease and no systemic infection was obtained at autopsy from the Department of Pathology of the VU University Medical Centre (VUmc), Amsterdam. As part of the patient contract, or if relatives have given explicit prior written consent, all tissue sections can be used for research after completion of the diagnostic process. The tissues were immediately fixed in 4% formalin and subsequently embedded in paraffin for analyses. The study was approved by the medical ethics committee of the VUmc, in accordance with the guidelines provided by the World Medical Association (Declaration of Helsinki).

### Viral genome analysis

Four 20-µm-thick paraffin-embedded formalin-fixed tissue sections were cut for isolation of DNA and mRNA. To each sample, 500 µl TRIS-HCl with 0.45% sodium dodecyl sulphate and 2 mg/ml proteinase K (Roche, Basel, Switzerland) were added, vortexed and incubated at 55 °C for 2 h. Next, the samples were incubated at 95 °C for 10 min and then centrifuged for 2 min at 17,000 *g*. DNA and mRNA were isolated from the supernatant using a QIAsymphony isolation robot (Qiagen, Hilden, Germany) following the manufacturer’s instructions. The DNA and mRNA isolates were stored at −80 °C. Polymerase chain reaction (PCR) analyses for the presence of parvovirus B19 (PVB19), Epstein-Barr virus (EBV), cytomegalovirus (CMV), human herpesvirus‑6 (HHV-6), adenovirus and enterovirus genomes was performed at the Virology Department of the Academic Medical Centre, Amsterdam, in accordance with established diagnostic protocols. Primer and probe details can be found in Tab. 1.

**Table 1:** Primers and probes used for quantitative polymerase chain reaction analysis

Target	Primer/probe	Sequence 5’- 3’
PVB19	Forward	CAC CCC CAT GCC TTA TCA
	Reverse	TGC CCA GGC TTG TGT AAG TCT
	Probe	TCA TGC AGA ACC TAG AGG AGA AAA TGC AGT ATT ATC T
EBV	Forward	CAC AAT GTC GTC TTA CAC CAT TGA
	Reverse	AGG TCC TTA ATC GCA TCC TTC A
	Probe	CGT CTC CCC TTT GGA ATG GCC C
CMV	Forward	CAA GCG GCC TCT GAT AAC CA
	Reverse	ACT AGG AGA GCA GAC TCT CAG AGG AT
	Probe	TGC ATG AAG GTC TTT GCC CAG TAC ATT CT
HHV‑6	Forward	TTT GCA GTC ATC ACG ATC GG
	Reverse	AGA GAG CGA CAA ATT GGA GGT TTC
	Probe	AGC CAC AGC AGC CAT CTA CAT CTG TCA A
Adenovirus	Forward	CAG GAC GCC TCG GRG TAY CTS AG
	Reverse	GGA GCC ACV GTG GGR TT
	Probe	CGG GTC TGG TGC AGT TTG CCC GC
Enterovirus	Forward	GGC CCT GAA TGC GGC TAA T
	Reverse	GGG ATT GTC ACC ATA AGC AGC C
	Probe	GCG GAA CCG ACT ACT TTG GGT

*PVB19* parvovirus B19, *EBV* Epstein-Barr virus, *CMV* cytomegalovirus,* HHV‑6* human herpesvirus 6

### Immunohistochemistry

Serial tissue sections (4 µm) were deparaffinised in xylene, dehydrated in 100% ethanol and endogenous peroxidase was blocked in methanol with 0.3% H_2_O_2_ for 30 min. Antigen retrieval was performed by heat inactivation in 10 mM Tris-EDTA buffer (pH 9.0; boiled for 10 min) for immunostaining of CD3. No antigen retrieval was required for CD45. Subsequently, tissue sections were incubated with either rabbit anti-human CD3 (1:50 dilution; Dako Agilent, Amstelveen, The Netherlands) or mouse anti-human CD45 (1:50 dilution; Dako Agilent) for 60 min at room temperature. After a wash in phosphate-buffered saline the slides were incubated with Envision HRP anti-mouse/anti-rabbit (undiluted, Dako) for 30 min. The slides were counterstained with haematoxylin, dehydrated and covered. For fibrosis analysis, tissue cross-sections were stained using the histological Elastica von Gieson (EvG) staining method, according to the standard protocol. Negative controls were included with each staining and showed no staining (not shown). All slides were scored by two independent observers (L. Wu and P.A.J. Krijnen; inter-observer variation <10%).

### Quantification of inflammatory cells

CD3+ and CD45+ cells were counted on serial tissue cross-sections using a light microscope (Zeiss, Germany, 250× magnification). The tissue surface areas were then measured using Qprodit v3.2 (Leica Microsystems, Rijswijk, The Netherlands) and the number of positive cells per mm^2^ was calculated. Only CD45+ cells with a round morphology, scant cytoplasm and a distinct peripheral reactivity were counted. In this way, the common leucocyte marker CD45, which is present on non-lymphocytic cells also, can be used as a general lymphocyte marker [2]. For the quantification of fibrosis, EVG-stained slides were scanned using a PathScan Enabler IV slide scanner (Meyer Instruments, Houston, TX, USA). The surface area of fibrosis within the atrial myocardium was measured using QuickPhoto Micro analysis software (Promicra, Prague, Czech Republic). The quantitative analyses were performed blinded.

### Statistical analysis

Statistical analysis was performed with SPSS (Windows version 2.0, IBM Corp., Armonk, NY, USA). The discrete variable was expressed as mean ± standard deviation (SD) unless stated otherwise. For non-normally distributed data, Mann-Whitney U tests were used. Putative differences in PVB19 prevalence were analysed using a chi-square test. *p*-values <0.05 were considered statistically significant.

## Results

### Patients

The clinical characteristics of the control (*n* = 14) and AF (*n* = 50) patients are depicted in Tab. 2. The control patients had an average age of 49 years, ranging from 29 to 81 years, and were significantly younger than the paroxysmal AF patients (average age 66 years, range 41–79 years; *p* < 0.001) and the long-standing persistent/permanent AF patients (average age 62 years, range 38–84 years; *p* < 0.01). Gender distribution and the prevalence of diabetes, recent myocardial infarction, angina pectoris and hypertension did not differ significantly between the three groups. More detailed clinical information on the AF patients is presented in Table S1 (Electronic Supplementary Material). Left atrial dimension, left ventricular ejection fraction, the prevalence of valve pathology, concomitant arrhythmias, left ventricular hypertrophy (LVH) as well as previous treatment did not differ significantly between AF subtypes.

**Table 2:** Patient characteristics (*n* = 64)

Measurement	Control (*n* = 14)	Paroxysmal AF (*n* = 20)	LS-PE/PER AF (*n* = 30)
Age (years), mean (±SD)	49 (±13.3)	66 (±10.2)^***^	62 (±12.2)^**^
Male/female	10/4 (71%/29%)	13/7 (65%/35%)	23/7 (77%/23%)
Diabetes mellitus	0 (0%)	4 (20%)	4 (13%)
*Cardiovascular disease*			
Recent myocardial infarction	0 (0%)	2 (10%)	2 (7%)
Angina pectoris	0 (0%)	2 (10%)	2 (7%)
Hypertension	3 (21%)	1 (5%)	1 (3%)

*AF* atrial fibrillation, *LS-PE/PER AF* long-standing persistent and permanent AF

***p < 0.001, **p < 0.01 compared with control group

### Analysis of viral genomes in the atria

To analyse a putative role of viral infection of the atria in the pathogenesis of AF, the presence of viral genomes was verified in atrial tissue of AF patients via PCR of six cardiotropic viruses that are most frequently found to be associated with myocarditis, namely PVB19, EBV, CMV, HHV‑6, adenovirus and enterovirus [12]. Five (10%) of 50 AF patients (paroxysmal, *n* = 2; long-standing persistent/permanent, *n* = 3) tested positive for PVB19, while the genome of the other viruses was not found. We subsequently established the presence of the PVB19 genome in the atria of 7 of 14 (50%) control patients. The PVB19 genome was significantly more prevalent in control than in AF patients (*p* < 0.01). LVH was significantly more prevalent in PVB19-positive (+) than PVB-negative (−) AF patients (*p* = 0.028), whereas none of the other clinical parameters differed significantly between these groups.

### Inflammation in PVB19+ versus PVB19− control and AF patients

**Fig. 1:**
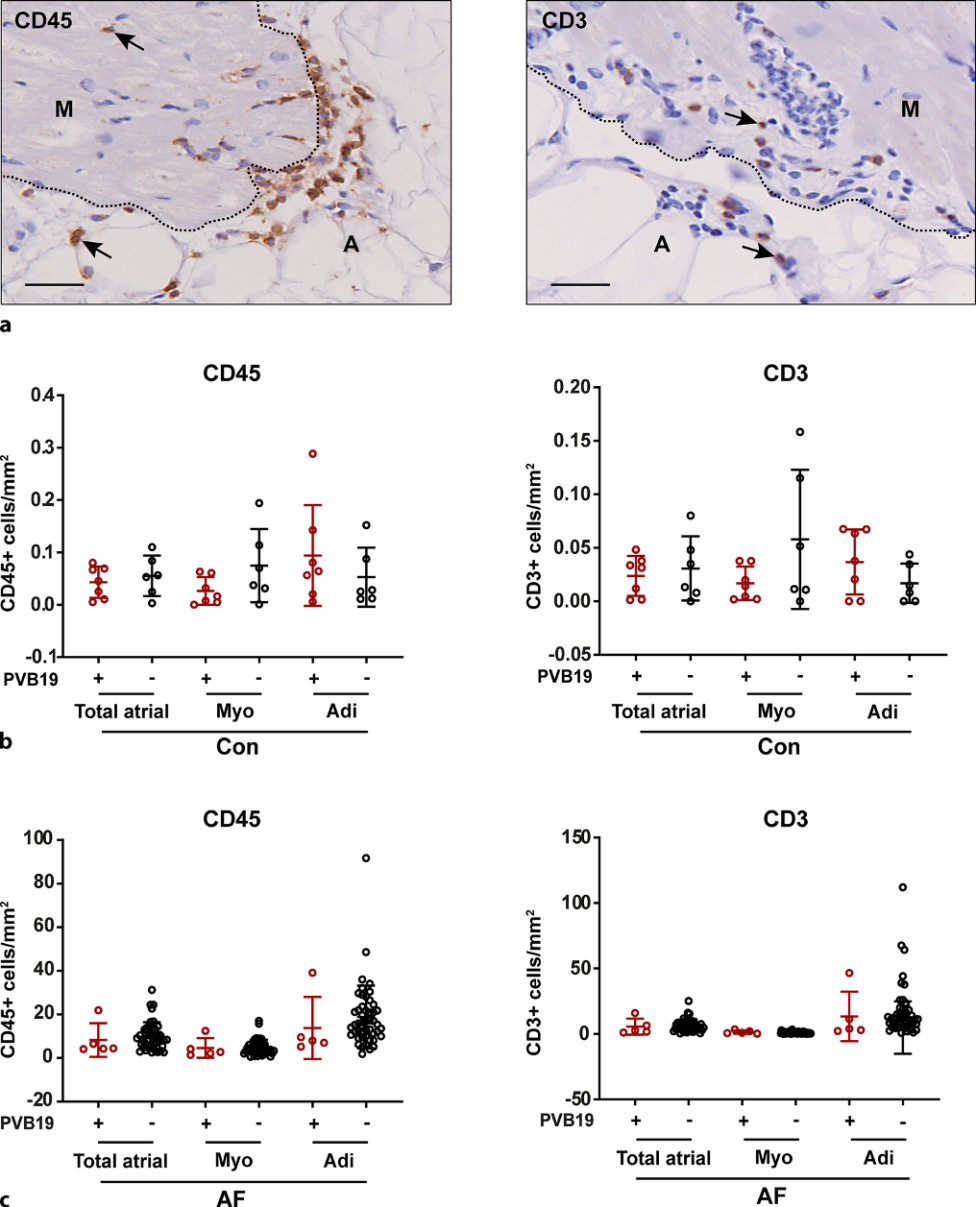
**a**–**c** The atrial inflammatory cell infiltrate in parvovirus B19 (*PVB19*)-positive versus PVB19-negative control and atrial fibrillation (*AF*) patients. **a** An example of CD45+ and CD3+ cells (*black arrows*) in the atria of patients with AF. *M* myocardium, *A* adipose tissue, *scale bar* = 50 μm. The number of CD45+ and CD3+ cells in the total atrial tissue and the myocardium (*Myo*) and adipose (*Adi*) tissue separately of **b** control (*Con*) patients and **c** AF patients that tested positive (PVB19+) or negative (PVB19−) for PVB19. Each *point* in the graphs represents the value of one individual patient; the *bars* represent mean ± SD

We wondered whether the presence of the PVB19 genome in the atria was associated with the extent of atrial inflammation. Hence we compared the atrial densities of CD3+ T lymphocytes and CD45+ lymphocytes between PVB19+ and PVB19− patients (Fig. 1). Some of the data related to atrial inflammation in these patients has been published previously [3]. As the number of PVB19+ AF patients was limited, these analyses were performed combining the PVB19+ paroxysmal and long-standing persistent/permanent AF patients. As expected and reported previously [3], the numbers of CD45+ and CD3+ cells in the atria of control patients (Fig. 1b) were significantly lower than in AF patients (Fig. 1c). However, both in control and AF patients no significant differences were observed in the numbers of CD45+ and CD3+ cells/mm^2^ in the total atrial tissue, nor in the myocardium and adipose tissue separately, between PVB19+ or PVB19- patients.

### Analysis of fibrosis in the atria

**Fig. 2:**
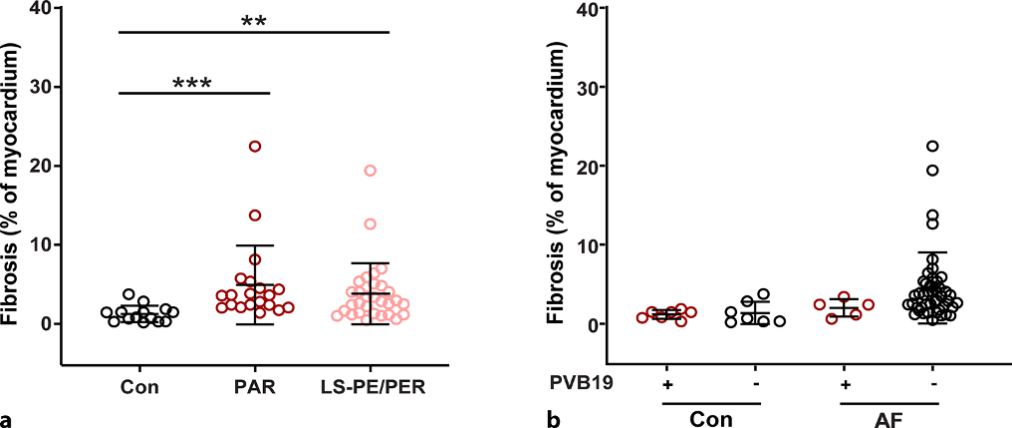
**a**, **b** The percentage of fibrosis in the atrial myocardium of control and atrial fibrillation (*AF*) patients. **a** The percentage of fibrosis in the atrial myocardium in control group patients without AF (*Con*), patients with paroxysmal AF (*PAR*) and patients with long-standing persistent/permanent AF (*LS-PE/PER*). **b** The percentage of fibrosis in the atrial myocardium in parvovirus B19 (*PVB19*)-positive and PVB19-negative control and AF patients. Each *point* in the graphs represents the value of one individual patient; the *bars* represent mean ± SD. **p < 0.01, ***p < 0.001

Previous research has shown that atrial inflammation can stimulate atrial fibrosis formation and increase the risk of AF development[13]. Therefore, we compared the percentage of fibrosis in the atrial myocardium of control and AF patients (Fig. 2). We found a significant increase in the area of fibrosis within the atrial myocardium of paroxysmal AF (*p* < 0.001) and long-standing persistent/permanent AF (*p* < 0.01) patients compared with the control group (Fig. 2a). However, we found no significant differences in atrial fibrosis between PVB19+ and PVB19− control or AF patients (Fig. 2b).

## Discussion

Previous observations support a possible role for viral infection in the development of AF [14, 15]. It is, however, unknown whether these viruses, of which many are cardiotropic, also directly infect the atria in AF. Therefore, in this study we analysed the presence of genomes of different cardiotropic viruses in atrial tissue from AF patients as evidence of (past) infection. Of the viruses tested, only the PVB19 genome was found in the atria of 10% of the AF patients. However, the PVB19 genome was also found in 50% of the control patients. Furthermore, we found no apparent association between the presence of the PVB19 genome and the extent of atrial inflammation or fibrosis in control and AF patients.

Of the different viruses tested only the PVB19 genome was found in the atria of 10% of the AF patients. However, the relevance of this genomic PVB19 presence in the pathogenesis of AF in these patients can be questioned. Although the PVB19 genome has been demonstrated in the myocardium of patients with virus-related heart disease [16, 17], it has also been found in the hearts of a high percentage (up to 96%) of patients without myocarditis or dilated cardiomyopathy [14, 18]. These studies have thus shown that the PVB19 genome can be latent and persistent life-long in the heart and do not necessarily indicate an active viral infection. Indeed, in our study we also found the PVB19 genome in the atria of 50% of the control patients, who showed no clinical and histological evidence of cardiac disease. It is nevertheless a theoretical possibility that the result of viral infection of the atria can differ between individuals and that this may relate to differences in the severity of the immune response and tissue damage. For instance, it is known that in some patients with viral myocarditis such a severe immune response can become autoimmune and continue to damage the heart even after viral clearance [12]. However, although atrial inflammation is increased in AF patients [2, 3], we found no association between the presence of the PVB19 genome and the extent of atrial inflammation or atrial fibrosis in our AF patients or in the controls. These results then do not support an active role for PVB19 in the pathogenesis of AF in these patients.

As with PVB19, the genomes of herpesviruses such as EBV, CMV, HHV‑6 and adenoviruses often establish latency after primary infection [15]. Furthermore, persistent and latent enterovirus genome without viral replication was detected in the hearts of patients with ischaemic or dilated cardiomyopathy [19]. The fact that we did not find genomic material of these viruses suggests the atria were not previously infected by these viruses. However, we cannot exclude the possibility that a prior viral infection was already cleared.

The increase in atrial inflammation and fibrosis we observed in AF patients compared to non-AF controls is in line with studies alluding to the role of atrial inflammation and fibrosis as an arrhythmic substrate. However, the similar amounts of inflammation and fibrosis in the PVB19+ and PVB19− patients further suggest, but do not prove, that PVB19 infection is not related to the pathophysiology of AF.

Lastly, cardiac surgery such as coronary artery bypass grafting and aortic valve replacement surgery may increase the risk of virus reactivation [20, 21]. However, as all the tissues used in this study were obtained as soon as possible after the start of the procedure, it is unlikely that this affected the results.

This study has some limitations. Both the control and AF patients were included retrospectively. Selection bias may exist in the AF group, as atrial tissue was available only from patients who underwent cardiac surgery, and this may not accurately reflect AF patients that do not require surgical intervention. In addition, information bias may exist as a result of incomplete patient data, especially in the autopsied control patients, and may be relevant for interpretation of the results. Other limitations are the relatively small number of patients and possible sampling error, i.e. that the results recorded in the atrial appendage may not fully represent other areas of the atria.

In conclusion, we found no increased presence of cardiotropic viral genomes in the atrial tissue of AF patients. All in all these results do not support an important role for viral infection of the atria in the pathogenesis of AF in our patient cohort.

## Supplementary Information

*Table S1* Clinical information of atrial fibrillation (*AF*) patients (*n* *=* 50)
