# Supplementary material for: The presence of cardiotropic viral genomes is not increased in atrial tissue of atrial fibrillation patients
Source: Neth Heart J. 2022 Jan 31;30(7-8):377–82. doi: 10.1007/s12471-022-01660-4 (PMC9270523; doi:10.1007/s12471-022-01660-4)
Supplement: Supplementary file 1 — Table S1 Clinical information of atrial fibrillation (AF) patients (n = 50) [file 12471_2022_1660_MOESM1_ESM.docx]

**Table S1** Clinical information of atrial fibrillation (*AF*) patients (*n=*50)

| **No** | **AF** | **Sex** | **Age** | **Concomitant cardiac surgery** | **Atrial dimension (ml/m^2^)** | **LVEF**  **(%)** | **LVH** | **Valve pathology** | **Arrhythmias** | **Previous AF treatment** | **Anti-arrhythmic drugs** |
| --- | --- | --- | --- | --- | --- | --- | --- | --- | --- | --- | --- |
| 1 | PAR | M | 58 | No CCS | 29.94 | 60 | No | No | No | Medical treatment | Flecainide |
| 2 | PAR | M | 76 | Valve surgery CABG | n.k. | 60 | No | Yes | No | Medical treatment | Sotalol, lanoxin |
| 3 | PAR | M | 74 | CABG | n.k. | 40 | Yes | Yes | No | Medical treatment | Sotalol |
| 4 | PAR | F | 69 | Valve surgery | 57.03 | 60 | No | Yes | No | Medical treatment | Metoprolol |
| 5 | PAR | F | 65 | Valve surgery | n.k. | 68.3 | No | Yes | No | Medical treatment | Metoprolol |
| 6 | PAR | M | 66 | CABG | 39.42 | 45 | No | No | No | Cardioversion, medical treatment | Metoprolol |
| 7 | PAR | F | 63 | CABG | 55.83 | 65 | No | No | No | Medical treatment | Sotalol |
| 8 | PAR | M | 70 | CABG | 56.33 | 60 | No | No | No | Cardioversion, medical treatment | Lanoxin |
| 9 | PAR | F | 48 | No CCS | 35.8 | 65 | No | No | No | Cardioversion, medical treatment | Amiodarone |
| 10 | PAR | F | 77 | Valve surgery | n.k. | 60 | Yes | No | No | Cardioversion, medical treatment | Sotalol |
| 11 | PAR | F | 66 | No CCS | n.k. | 60 | No | No | No | Cardioversion, medical treatment | Flecainide |
| 12 | PAR | M | 76 | No CCS | n.k. | 60 | No | No | No | Cardioversion, medical treatment | Verapamil |
| 13 | PAR | M | 55 | No CCS | 38 | 60 | No | No | No | Cardioversion, medical treatment | Metoprolol |
| 14 | PAR | M | 74 | CABG | n.k. | 50 | No | Yes | No | Medical treatment | Metoprolol, lanoxin |
| 15 | PAR | M | 56 | CABG | 27.07 | 60 | No | No | No | Medical treatment | Sotalol |
| 16 | PAR | M | 65 | CABG | 38.51 | 60 | No | No | No | Medical treatment | Flecainide |
| 17 | PAR | M | 67 | No CCS | 42.89 | 55 | n.k. | No | No | Medical treatment | Flecainide |
| 18 | PAR | M | 76 | CABG | 75.36 | 60 | n.k. | No | No | Medical treatment | Metoprolol |
| 19 | PAR | F | 79 | Valve surgery | 33.8 | 53 | n.k. | Yes | No | No treatment | No drugs |
| 20 | PAR | M | 41 | No CCS | 31.68 | 71 | n.k. | No | No | Cardioversion, medical treatment | Metoprolol, flecainide |
| **No** | **AF** | **Sex** | **Age** | **Concomitant cardiac surgery** | **Atrial dimension (ml/m^2^)** | **LVEF (%)** | **LVH** | **Valve pathology** | **Arrhythmias** | **Previous AF treatment** | **Anti-arrhythmic drugs** |
| 1 | LS-PE/PER | M | 52 | CABG | 52.02 | 60 | No | No | No | Medical treatment | Flecainide |
| 2 | LS-PE/PER | M | 72 | No CCS | 33.28 | 35 | No | Yes | No | Medical treatment | Metoprolol, lanoxin |
| 3 | LS-PE/PER | M | 38 | No CCS | 20.2 | 20 | No | Yes | arrhythmias | Medical treatment | Metoprolol |
| 4 | LS-PE/PER | M | 55 | No CCS | n.k. | 60 | No | No | No | Medical treatment | Metoprolol, flecainide |
| 5 | LS-PE/PER | M | 48 | No CCS | 48 | 60 | No | No | No | Cardioversion, medical treatment | Flecainide |
| 6 | LS-PE/PER | F | 60 | CABG | n.k. | 60 | No | No | No | Medical treatment | Flecainide |
| 7 | LS-PE/PER | M | 65 | CABG | 36.99 | 60 | No | No | No | Medical treatment | Metoprolol |
| 8 | LS-PE/PER | M | 58 | No CCS | 26.67 | 60 | No | No | No | Medical treatment | Verapamil, flecainide |
| 9 | LS-PE/PER | M | 74 | CABG | n.k. | 31 | No | Yes | No | Medical treatment | Metoprolol, lanoxin |
| 10 | LS-PE/PER | F | 46 | No CCS | n.k. | 60 | No | No | No | Medical treatment | Metoprolol |
| 11 | LS-PE/PER | F | 69 | No CCS | 68 | 60 | No | No | No | Cardioversion, medical treatment | Amiodarone |
| 12 | LS-PE/PER | M | 58 | No CCS | n.k. | 60 | No | No | No | Medical treatment | Sotalol |
| 13 | LS-PE/PER | M | 67 | No CCS | 35.15 | 60 | No | Yes | No | Cardioversion, medical treatment | Sotalol |
| 14 | LS-PE/PER | M | 75 | Valve surgery CABG | 48.05 | 67 | No | Yes | No | Medical treatment | Metoprolol |
| 15 | LS-PE/PER | M | 56 | No CCS | 28.75 | 73 | No | No | No | Medical treatment | No drugs |
| 16 | LS-PE/PER | M | 56 | No CCS | 53.66 | 60 | No | No | No | Cardioversion, medical treatment | Metoprolol, lanoxin |
| 17 | LS-PE/PER | M | 67 | No CCS | 39.91 | 60 | No | No | No | PVI, medical treatment | Disopyramide |
| 18 | LS-PE/PER | F | 76 | Valve surgery  CABG | 32.47 | 27 | No | Yes | No | Medical treatment | No drugs |
| 19 | LS-PE/PER | M | 78 | Valve surgery CABG | 40.2 | 60 | No | No | No | PVI, medical treatment | Metoprolol, flecainide |
| 20 | LS-PE/PER | M | 80 | CABG | 39 | 45 | No | No | No | Medical treatment | No drugs |
| 21 | LS-PE/PER | M | 50 | Valve surgery | 46.96 | 44 | n.k. | Yes | arrhythmias | Medical treatment | Digoxin |
| 22 | LS-PE/PER | M | 54 | No CCS | 42.52 | 60 | n.k. | No | No | Cardioversion, medical treatment | Flecainide |
| 23 | LS-PE/PER | M | 84 | Valve surgery CABG | n.k. | 45 | n.k. | Yes | No | Medical treatment | Digoxin |
| 24 | LS-PE/PER | M | 73 | CABG | n.k. | 25 | n.k. | No | No | Medical treatment | Digoxin |
| 25 | LS-PE/PER | M | 40 | No CCS | 28.01 | 58 | n.k. | No | No | Cardioversion, medical treatment | Sotalol |
| 26 | LS-PE/PER | F | 73 | Valve surgery CABG | n.k. | 60 | n.k. | Yes | No | Medical treatment | Digoxin |
| 27 | LS-PE/PER | F | 50 | No CCS | 42 | 80 | n.k. | No | No | Cardioversion, medical treatment | Amiodarone |
| 28 | LS-PE/PER | M | 73 | Valve surgery CABG | n.k. | 50 | n.k. | Yes | No | Medical treatment | Metoprolol |
| 29 | LS-PE/PER | F | 58 | No CCS | 31.38 | 60 | n.k. | Yes | No | Medical treatment | Sotalol |
| 30 | LS-PE/PER | M | 66 | No CCS | n.k. | 60 | n.k. | No | No | Cardioversion, medical treatment | Sotalol |

*PAR* paroxysmal AF, *LS-PE/PER* long-standing persistent and/or permanent AF, *LVEF* left ventricle ejection fraction, *LVH* left ventricle hypertrophy, *CCS* concomitant cardiac surgery, *CABG* coronary artery bypass grafting, *PVI* pulmonary vein isolation, *n.k*. not known
